# Anti-aging dilemma: to restore the hardware or to reinstall the software?

**DOI:** 10.3389/fgene.2015.00129

**Published:** 2015-04-14

**Authors:** Ancha Baranova, James D. Willett

**Affiliations:** ^1^Center for the Study of Chronic Metabolic Diseases, School of Systems Biology, George Mason UniversityFairfax, VA, USA; ^2^Research Centre for Medical GeneticsMoscow, Russia

**Keywords:** metabolome, SEUs, mutations, Turing machines, hypercomputation

“Metaphors have a way of holding the most truth in the least space.”

—Orson Scott Card, *Alvin Journeyman*

Since its early days, the value of deciphering the human DNA has been seen primarily in extracting the set of messages that run the cells that constitute the body. In common understanding, these messages are encoded in DNA and transcribed as cell-specific sets of RNAs, some of which are translated to proteins, then modified with various post-translational add-ons made of sugars, lipids and other moieties. This complex chain of events is further complicated by multi-layered possibilities for the modifications allowed at every step–epigenetics for DNA, editing for RNAs and the recently discovered phenomenon of non-template polypeptide extension allowed by ribosomes (Shen et al., 2015). It seems that when looked at as a whole, the DNA, and all the messages associated with DNA, do not look like a blueprint, or even a clear set of instructions, but rather a messy draft or a stack of notes that are scribbled all over and full of ambiguities.

However, let us hack through the majority of the “omics” and look upon the set of small molecules known as metabolites, and the budding discipline of Metabolomics that researches the true underpinnings of the abundantly complex mechanics of the living cell. It is worthwhile to note that, to a somewhat defined degree, the cell will tolerate the loss of a gene or changes in the levels of RNAs or even the most important of proteins, while even slight deregulation of the levels of some of the smallest metabolites leads to immediate and catastrophic consequences. The potassium ion and ATP may be used as the primary examples of smallest molecules capable of eliciting a systemic response. According to our calculation, a mere 0.5% increase in the total content of potassium chloride, one of the most common electrolytes in the human body, leads to immediate cardiac arrest. The consequences of the depletion of ATP may manifest as a variety of ailments, with their duration inversely proportional to the severity of the defect. Aging, in particular, is associated with a decline in the efficiency of oxidative phosphorylation and an increase in the risk of resulting pathologies. Of course, there are other small molecules, possibly not as well-known as ATP, but still indispensable and irreplaceable. In particular, the metabolites derived from the amino acid tryptophan have the capacity for similar dramatic alteration of system-wide function. Most pertinent to the topic of this discussion, the changes in metabolic profiles are considered as drivers for the pathogenesis of age-associated disorders, including Alzheimer's disease (Tacutu et al., 2010; Demetrius and Driver, 2013; Jia et al., 2014; Obre and Rossignol, 2015). It also is of note that metabolites are not as abundant as commonly studied species of proteins and RNAs. Hence, the world of metabolites is immensely easier to comprehend than the overly complicated world of other famous “omics.” The latter point is extremely important, as it provides a possibility for the use of a powerful reductionist approach without falling into ill-fitting or over-fitting of the underlying model, a well-known perpetual source of entrapment.

Let us compare the living (and aging) cell, with its endless “omics”-scale layers of interconnected components, to modern computers. Computer hardware is a collection of interconnected physical devices used in or with your machine. One of these parts may wear itself out and die; however, in industrially–built computers, the presence of multiple redundant circuits incorporated in fault-tolerant or multi-modular redundancy designs provides adequate protection against so-called “**soft errors**.” But what are such soft errors? Indeed, these are not synonymous with “software glitches.” Soft errors are often defined as “**single-event upsets**” (SEU), the changes of state caused by ions or electro-magnetic radiation striking a sensitive node in a micro-electronic device, usually a unit where the memory is stored. The most common cause of “soft errors” is the direct hit of the circuits by cosmic particles colliding with atoms in the atmosphere, creating cascades or showers of neutrons and protons (Ziegler and Lanford, 1979)–analogous to the occurrence of a mutation. There is even a formula for the calculation of a soft-error rate that is typically expressed as a number of failures-in-time (a.k.a. the mutation rate) (Li et al., 2007). Similarly to living systems, computers could be and are designed to detect SEUs and recover gracefully, either by forward error correction that incorporates redundant error-correcting code into each output, or by roll-back error correction that detects the SEU using “sentinel” (or parity) bits, and, if needed, rewrites the data using a back-up. In the DNA world, both of these functions are executed by the DNA repair machinery. Similarly to redundant routines fixing SEUs, the redundant DNA repair mechanisms are embedded in the original system's design, whether *in vivo* or *in silico*. Hence, both the DNA and the molecules directly encoded by DNA, i.e., the RNA and the proteins, are the components of the hardware of life.

The million dollar question is: “What components make up the living cell's software?” Below we will try to make a case for the community of small molecules extant within the metabolome as the software components that run the living cell. Indeed, metabolites are universal, and also interchangeable between cellular types. In the end, ATP is only ATP; it is difficult to imagine that ATP may be mutated into something else. Hence, the metabolites may be likened to the set of instructions (software), that could be run on one or another type of hardware–i.e., a molecule of ATP extracted from a tapeworm would have addressed cellular needs and functions in the same way as that extracted from a human cell. Within the cell, a set of metabolites, each with its associated local concentrations and, possibly, their ratios may serve as the “net regulator” directing the overall patterns of transcription, translation and further modification of the messages encoded in DNA. Importantly, the concentrations of metabolites may be adjusted externally, either through direct supplementation or the administration of soluble enzyme inhibitors or co-factors. With that, very similarly to computer software, the “net regulation” that is maintained by the cellular metabolome may be restored to the default settings. In the case of aging, the default mode would correspond to one or another earlier timepoints on the living system trajectory, or the “younger” state of the living system. As one of us has previously demonstrated, metabolic profiles are robust, reproducible fingerprints of whole organismal phenotypic states in the nematode, *Caenorhabditis elegans*, accurately reflecting both life stage differentials and environmental modulations (Willett et al., 2010; Sudama et al., 2013). Perhaps it is more than a coincidence that Sydney Brenner, a founding father of the use of *C. elegans* as a transparent model for various scientific inquiries, including aging research, recently pointed at the biological necessity of including the question of information into the eternally studied interplay between matter and energy (Brenner, 2012).

No doubt, all of the above are nothing more than metaphors. However, these analogies may be helpful for understanding the eternal problem of aging as the mundane crackdown of a desktop computer. When the desktop is starting to fail us by slowing down or freezing frames, we either reboot it or, as a last resort, reinstall its operating software. Note that the idea of redesigning or otherwise reinforcing the hardware parts to make them less prone to SEUs, or mutations, in the case of a desktop seems absurd. Similarly, as a countermeasure to aging, we should concentrate on the elements that are easily fixable, or replaceable–the metabolic pattern seems like a suitable candidate for extrinsic or intrinsic modifications (Muradian, 2013). Obviously, this avenue of thinking implies that aging is not a fundamental property of the living system, but rather a time-associated decay, and that one or another routine procedure may be established in order to offset this process, in a way that is quite similar to the treatment of the disease.

In other words, the desktop metaphor provides for a hope that a software ingredient of the living machine, the metabolism, may be amenable to rebooting. Indeed, the analogies between the computer world and the world of living (and aging) things are abundant. Since the beginning of modern science, physics and its extension, chemistry, have been considered to be the foundation of biology. In digital physics, all known laws of physics have consequences that are theoretically computable on a digital computer, and therefore the universe itself must be computable on a classical Turing machine, a hypothetical device that manipulates symbols on a strip of tape according to a table of rules (Turing, 1936). The essential truth postulated above is known as the Strong Church–Turing thesis (Copeland, 1996). Pertinent to biology, living systems are parts of the Turing Universe; hence, all living things are Turing computers and, therefore, are the subjects of biological determinism in the widest sense possible.

While giving both the grounds for an infinite number of scientific papers describing various mechanistic insights into “regulatory” cellular networks and providing hope for the ultimate understanding of living system trajectories, we must admit that digital physics is neither the most modern, nor the most attractive representation of the universe. There are some widely-discussed alternatives, for example, that the Universe is a hypercomputer that is capable of non-recursive calculations (Siegelmann, 1995; Copeland and Proudfoot, 1999).

Importantly, even if the Universe as a whole may be likened to a hypercomputer, it is possible that its parts, i.e., living systems, may firmly remain within the Turing realm. Here we would like to add to a recent argument that makes the case of living systems surpassing the Turing requirements (Maldonado and Gomez Cruz, 2015) by referring to the Turing-unsolvable halting problem. An inability to detect a halt or, in other words, to determine from a description of an arbitrary computer program and an input whether the program will finish running, or continue to run forever, is a feature embedded in a Turing design (Jack Copeland, 2004). Here we postulate that, for the living system, death is equivalent to a halt. Since in living systems one can both detect and predict death with certainty, we should accept non-recursive hypercomputation as an important underlying principle of biology.

## Conflict of interest statement

The authors declare that the research was conducted in the absence of any commercial or financial relationships that could be construed as a potential conflict of interest.
